# Editorial Brevities

**Published:** 1887-06

**Authors:** 


### EDITORIAL BREVITIES.

DR. DANIEL E. BRONTON, so long connected with the *Medical and Surgical Reporter*, Philadelphia, has retired from that journal.

DR. JOSEPH JONES, of New Orleans, was elected President of the Louisiana Medical Society for the ensuing year. A good selection, truly.

A SCHOOL OF MEDICINE for Women in Russia was recently closed after being in existence a period of fourteen years.

SEE advertisement of the Dental and Medical Departments of the Southern Medical College.

WELLS & RICHARDSON—We invite special attention to the advertisement of the above house appearing for the first time in the present issue of our journal.

E. A. YARNALL—SURGICAL INSTRUMENTS.—A new advertisement from this excellent house relating to surgical instruments appears in this issue, which our readers should examine.

WE invite attention to Liebig's Liquid Food, as in advertisement on page eleven of advertising department. It is a valuable preparation of concentrated beef containing both nutritive and tonic properties. Note, also, their Cherry Malt Phosphates.

REID & CARNRICH.—We invite attention to the new advertisement of this old and reliable house, in our present issue. Now that the season for the Summer complaints of children is upon us the profession will find in the advertisement referred to something timely and appropriate.

THE OBSTETRIC SECTION AT THE INTERNATIONAL CONGRESS.—Parties desiring to contribute to the section on Obstetrics are requested to send their papers to Prof. DeLaskie Miller, of Chicago, Ill., the President of the Section, or to Prof. T. S. Powell, of Atlanta, Ga., Vice President of the same

THERE are said to be two thousand more doctors in the State of Illinois than are needed.

WARM ETHER.—It is now recommended to warm ether before using as an anæsthetic. It acts more promptly and efficiently. Don't forget, however, that it is very inflammable.

DR. ROBERT KOCH.—That indefatigable house of Detroit—Parke, Davis & Co.—kindly sends us a large and beautifully executed portrait of the celebrated Dr. Robert Koch, the renowned discoverer of the Bacielus Tuberculosis. We highly appreciate the picture, and warmly thank Messrs. Parke, Davis & Co. for the same.

